# Symbiotic Growth of a Thermophilic Sulfide-Oxidizing Photoautotroph and an Elemental Sulfur-Disproportionating Chemolithoautotroph and Cooperative Dissimilatory Oxidation of Sulfide to Sulfate

**DOI:** 10.3389/fmicb.2019.01150

**Published:** 2019-05-24

**Authors:** Shigeru Kawai, Naoki Kamiya, Katsumi Matsuura, Shin Haruta

**Affiliations:** Department of Biological Sciences, Tokyo Metropolitan University, Hachioji, Japan

**Keywords:** anoxygenic photosynthesis, sulfur-disproportionation, hot spring microbial mats, sulfide, elemental sulfur

## Abstract

A thermophilic filamentous anoxygenic photosynthetic bacterium, *Chloroflexus aggregans*, is widely distributed in neutral to slightly alkaline hot springs. Sulfide has been suggested as an electron donor for autotrophic growth in microbial mats dominated with *C. aggregans*, but remarkable photoautotrophic growth of isolated *C. aggregans* has not been observed with sulfide as the sole electron source. From the idea that sulfide is oxidized to elemental sulfur by *C. aggregans* and the accumulation of elemental sulfur may have an inhibitory effect for the growth, the effects of an elemental sulfur-disproportionating bacterium that consumes elemental sulfur was examined on the autotrophic growth of *C. aggregans*, strain NA9-6, isolated from Nakabusa hot spring. A sulfur-disproportionating bacterium, *Caldimicrobium thiodismutans* strain TF1, also isolated from Nakabusa hot spring was co-cultured with *C. aggregans. C. aggregans* and *C. thiodismutans* were successfully co-cultured in a medium containing thiosulfate as the sole electron source and bicarbonate as the sole carbon source. Quantitative conversion of thiosulfate to sulfate and a small transient accumulation of sulfide was observed in the co-culture. Then the electron source of the established co-culture was changed from thiosulfate to sulfide, and the growth of *C. aggregans* and *C. thiodismutans* was successfully observed with sulfide as the sole electron donor for the autotrophic growth of the co-culture. During the cultivation in the light, simultaneous consumption and accumulation of sulfide and sulfate, respectively, were observed, accompanied with the increase of cellular DNAs of both species. *C. thiodismutans* likely works as an elemental sulfur scavenger for *C. aggregans*, and *C. aggregans* seems to work as a sulfide scavenger for *C. thiodismutans*. These results suggest that *C. aggregans* grows autotrophically with sulfide as the electron donor in the co-culture with *C. thiodismutans*, and the consumption of elemental sulfur by *C. thiodismutans* enabled the continuous growth of the *C. aggregans* in the symbiotic system. This study shows a novel symbiotic relationship between a sulfide-oxidizing photoautotroph and an elemental sulfur-disproportionating chemolithoautotroph via cooperative dissimilatory sulfide oxidation to sulfate.

## Introduction

The thermophilic filamentous anoxygenic photosynthetic bacterium, *Chloroflexus aggregans*, in the phylum *Chloroflexi*, is widely distributed in neutral to slightly alkaline hot springs (Hanada, 2003). This bacterium grows optimally at temperatures from 50 to 60°C. It is capable of growing photoheterotrophically under anaerobic conditions in the light and chemoheterotrophically under aerobic conditions in the dark (Hanada et al., 1995). Genome analysis of *C. aggregans* MD-66^T^ found a gene set for carbon fixation pathway suggesting that *C. aggregans* can grow autotrophically (Klatt et al., 2007). However, no photoautotrophic growth has been reported in *C. aggregans*, despite that photoautotrophic growth with sulfide or H_2_ as the electron source has been reported in other species in the genus *Chloroflexus*, *Chloroflexus* *aurantiacus* OK-70-fl (Holo and Sirevag, 1986) and *Chloroflexus* sp. strain MS-G (Thiel et al., 2014). Recently, we isolated new strains of *C. aggregans* from Nakabusa hot springs in Nagano, Japan, and found that some grew photoautotrophically and chemolithotrophically with H_2_ as an electron source (S. Kawai, A. Nishihara, K. Matsuura, and S. Haruta, manuscript in preparation). However, no strains of *C. aggregans* showed marked photoautotrophic growth with sulfide.

*Chloroflexus* spp. are generally thought to heterotrophically grow on organic compounds produced by cyanobacteria (van der Meer et al., 2005). In sulfidic hot springs, however, *Chloroflexus* spp. including *C. aggregans* are found to form microbial mats in the absence of cyanobacteria (Giovannoni et al., 1987; Kubo et al., 2011; Everroad et al., 2012; Otaki et al., 2012). Microbial communities dominated by *C. aggregans* form well-developed microbial mats in sulfidic hot spring water (∼0.1 mmol L^−1^ sulfide) at Nakabusa hot springs (Kubo et al., 2011; Everroad et al., 2012; Otaki et al., 2012). Since organic compounds and H_2_ in the hot spring water are scarce (Nakagawa and Fukui, 2002), it has been the presumption that *C. aggregans* grows photoautotrophically using sulfide as the major electron source in the hot springs. A previous study showed bicarbonate-dependent sulfide-oxidation in the *C. aggregans*-dominated microbial mats under anaerobic conditions in the light (Kubo et al., 2011). In addition, the *C. aggregans* genome contains the sulfide:quinone oxidoreductase gene for sulfide oxidation, but lacks dissimilatory sulfite reductase genes (Holkenbrink et al., 2011) and a gene set for the SOX pathway (Klatt et al., 2007). These observations suggest that *C. aggregans* can grow photoautotrophically through oxidation of sulfide to elemental sulfur under anaerobic conditions.

Concerning the difficulty of the observation of photoautotrophic growth on sulfide in *C. aggregans*, we hypothesized that accumulation of the oxidized product of sulfide, i.e., elemental sulfur, may suppress autotrophic growth of *C. aggregans*, and the removal of the elemental sulfur by other bacterial species may be required to stimulate autotrophic growth. Antibacterial effects of elemental sulfur have been known in various species of bacteria (Libenson et al., 1953; Pestana and Sols, 1970). One possible consumer of elemental sulfur, the sulfur-disproportionating bacterium, *Caldimicrobium thiodismutans* TF1, has been isolated by Kojima et al. (2016) from Nakabusa hot spring. *C. thiodismutans* TF1 grows autotrophically on elemental sulfur and thiosulfate to produce sulfide and sulfate under anaerobic conditions in the dark when ferrihydrite is added as a sulfide scavenger to support the growth. Identical 16S rRNA sequences to that of *C. thiodismutans* TF1 have been detected from the microbial communities with *C. aggregans* (Everroad et al., 2012).

In this study, we established a co-culture of *C. aggregans* and *C. thiodismutans* with sulfide as the sole electron source under autotrophic conditions. We evaluated the growth of *C. aggregans* when supported by the growth of *C. thiodismutans* consuming elemental sulfur, which would be produced by photo-anaerobic oxidation of sulfide by *C. aggregans*. Before the establishment of the co-culture on sulfide, co-cultivation on thiosulfate was conducted to start a co-culture without ferrihydrite, a sulfide scavenger, that was required for the growth of *C. thiodismutans* in axenic culture.

## Materials and Methods

### Bacterial Strains and Cultivation Conditions

*C. aggregans* strain NA9-6 was isolated from Nakabusa hot spring, Japan (S. Kawai, A. Nishihara, K. Matsuura, and S. Haruta, manuscript in preparation). This strain shows good photoautotrophic growth with H_2_ as the electron donor and 98.7% identity of 16S rRNA gene sequence with the type strain of *C. aggregans* (MD-66 = DSM 9485^T^). *C. aggregans* NA9-6 was cultivated in a medium lacking an organic carbon source. The autotrophic medium was composed of (L^−1^) 0.38 g KH_2_PO_4_, 0.39 g K_2_HPO_4_, 0.5 g NH_4_Cl, 4.2 g NaHCO_3_, and 0.5 g Na_2_S_2_O_3_⋅5H_2_O, buffered to pH 7.5. 5 ml of basal salt solution and 1 ml of vitamin mixture (Hanada et al., 1995) were added. Bacteria were cultivated at 55°C in the light (incandescent lamp; 2∼3 μmol m^−2^⋅s^−1^) under an H_2_:N_2_:CO_2_ (24:56:20, v:v:v) atmosphere. *C. thiodismutans* strain TF1 (Kojima et al., 2016), kindly provided by Dr. Fukui and Dr. Kojima, was cultivated in the dark at 70°C in the same autotrophic medium with added 2 mmol L^−1^ ferrihydrite under N_2_:CO_2_ (80:20) atmosphere. Ferrihydrite was prepared as previously described (Straub et al., 2005).

### Effects of Sulfide and Elemental Sulfur on the Growth of *C. aggregans* NA9-6

*C. aggregans* cells, cultivated in the autotrophic medium containing thiosulfate and H_2_ as described above, were collected by centrifugation, washed three times with the autotrophic medium and inoculated into 10 ml of the autotrophic medium in 30 ml test tubes. The gas phase of these culture tubes was H_2_:N_2_:CO_2_ (24:56:20, v:v:v). The initial optical density (OD) at 610 nm was adjusted to be 0.03. 0.05 g of sublimed sulfur (Wako, Osaka, Japan) sterilized at 110°C for 1 h three times or 1 mmol L^−1^ of sulfide was aseptically added into the medium to evaluate the effect of each compound on the growth. The inoculated tubes were cultivated at 60°C in the light (incandescent lamp; 2∼3 μmol m^−2^⋅s^−1^). OD at 610 nm of the culture tube was periodically measured during cultivation. Measurements were made after allowing tubes to stand at 60°C for 5 min in the dark to precipitate sulfur globules.

### Co-cultivation of *C. aggregans* and *C. thiodismutans* With Thiosulfate

*C. aggregans* NA9-6 and *C. thiodismutans* TF1 were separately cultivated under autotrophic conditions as described above. The two cultures were inoculated together into 5 ml of the autotrophic medium containing thiosulfate as a sole electron source under an N_2_:CO_2_ (80:20) atmosphere in 30 ml glass test tubes, capped with butyl rubber stoppers and screw caps. The tubes were cultivated at 60°C in the light (incandescent lamp; 2∼3 μmol m^−2^⋅s^−1^) for 7–20 days. 1 ml of the culture was repetitively subcultured in 5 ml of fresh medium under the same conditions to remove residual ferrihydrite from the original culture of *C. thiodismutans*. OD of the cultures was measured with a photometer (Colorimeter ANA18^+^, Tokyo photoelectric, Tokyo, Japan). 0.3 ml of the culture solution was sampled during cultivation for measurements of the amount of sulfur compounds in the culture.

### Co-cultivation of *C. aggregans* and *C. thiodismutans* With Sulfide

After repetitive subcultures of the co-culture of *C. aggregans* NA9-6 and *C. thiodismutans* TF1 in the medium containing thiosulfate, 1 ml of the co-culture solution was inoculated into 5 ml of the autotrophic medium containing 1 mmol L^−1^ of Na_2_S instead of thiosulfate in 30 ml glass test tubes, capped with butyl rubber stoppers and screw caps. The gas phase of the tube was N_2_:CO_2_ (80:20). The initial OD at 610 nm was adjusted to 0.03. The tubes were cultivated at 60°C in the light (incandescent lamp; 2∼3 μmol m^−2^⋅s^−1^). 0.3 ml of the culture solution was sampled during cultivation for measurements of the amount of sulfur compounds in the culture and extraction of DNAs from the cells.

### Measurements of Sulfur Compounds

Sulfide concentration in culture solution was measured using a methylene blue formation method as described previously (Cline, 1969) with some modifications. 50 μl of culture solution was mixed with 250 μl of 0.1 mmol L^−1^ carbonate-bicarbonate buffer (pH 10.0) immediately after collecting from the culture tube to prevent the loss of hydrogen sulfide. The mixture was centrifuged at 19,600 × *g* for 2 min at 4°C to remove bacterial cells. 200 μl of the supernatant was transferred to 400 μl of solution A (18 mmol L^−1^ zinc acetate, 0.2% v/v acetic acid) and stored at 4°C until measurements were made. The solution was alkalized by adding 400 μl of 40 mmol L^−1^ NaOH. After centrifugation of the mixture at 19,600 × *g* for 2 min, the precipitate was solubilized in 552 μl of solution A. Finally, 48 μl of Cline reagent (16 g of *N*,*N*-dimethyl-*p*-phenylenediamine sulfate, 24 g of FeCl_3_⋅6H_2_O in 1 L of 50% HCl) was added and mixed well. After 20 min incubation at room temperature, the absorbance at 665 nm was measured with a spectrophotometer (Infinite 200 PRO, Tecan, Seestrasse, Switzerland).

Thiosulfate and sulfate concentrations were determined by ion chromatography. 100 μl of culture was diluted with 900 μl of MilliQ water and centrifuged at 19,600 × *g* for 2 min. The supernatant was filtrated with a 0.45-μm-pore-size membrane filter. The solution was analyzed with a suppressed anion-exchange chromatography system (Shimadzu, Kyoto, Japan) equipped with a LC-20AD liquid chromatography, a DGU-20A_3_ degasser, a SIL-10AF auto sampler, a CT-20AC SP column oven, a CDD-10A SP conductivity detector, and a SCL-10A VP system controller. A Shim-pack IC-SA2 (250 mm × 4.0 mm; Shimadzu) was employed for separation of anions. The conditions of analyses were as follows; column temperature, 30°C; injection volume, 50 μl; eluent, 12 mmol L^−1^ NaHCO_3_ + 0.6 mmol L^−1^ Na_2_CO_3_; flow rate, 1.0 ml min^−1^. Data were collected and analyzed using Smart Chrome (KYA Technologies, Tokyo, Japan).

### DNA Extraction From Bacterial Cells

DNA was extracted using the benzyl chloride method (Zhu et al., 1993) combined with bead-beating. In brief, bacterial cells collected from culture by centrifugation were mixed with DNA extraction buffer (125 mmol L^−1^ Tris-HCl, 50 mmol L^−1^ EDTA, pH 8.0), SDS, and benzyl chloride. Culture cell suspensions were prepared in 2 ml tubes and incubated at 50°C for 2 h with mixing at 5 min intervals. Next, approximately 0.1 g of 0.1 mm diameter zirconia-silica beads (Biospec Products, Bartlesville, OK, United States) were added and the beating treatment was performed twice for 1 min at 2,500 rpm using a Mini-beadbeater (Biospec Products). DNA was recovered by phenol and chloroform extraction followed by alcohol precipitation with Ethachinmate (Nippon Gene, Tokyo, Japan). Finally, DNA was solubilized in TE buffer (10 mmol L^−1^ Tris-HCl, 1 mmol L^−1^ EDTA, pH 8.0) and stored at −20°C until use.

### Quantitative PCR Analysis

PCR primer sets were designed to distinguish 16S rRNA genes of *C. aggregans* and *C. thiodismutans*: agg-F (5′-CAAACGTGGTCTCAGTGCAGATCGG-3′) and agg-R (5′-TTAGCACACGGACTTCAAGCATTAG-3′) for *C. aggregans*; C-CmicF (5′-TACAATGGGGGGTACAGAGG-3′) and C-CmicR (5′-TGAGATAGCGACTTCGGGTG-3′) for *C. thiodismutans.* Specific amplification using each primer set was confirmed by DNA sequence analyses of the PCR fragments obtained using a mixture of both genomic DNAs. StepOne Real-Time PCR system (Applied Biosystems, Foster City, CA, United States) was used with FastStart Universal SYBR Green Master (Roche, Manniheim, Germany). The reaction mixture was composed of 10 μl of FastStart SYBR Green Master, 1 μl of 10 μM primers, 7 μl of water and 1 μl of DNA solution. Real-time PCR was performed under the following conditions; initial denaturation at 95°C for 10 min followed by 40 cycles of 15 s at 95°C and 1 min at 60°C. Fluorescence was detected at the end of the extension reaction. The purified DNA fragments amplified using the primer sets, agg-F and agg-R or C-CmicF and C-CmicR for the respective strain were spectrophotometrically quantified using Biospec-nano (Shimazu) and used as a template DNA to obtain the standard curve. The copy numbers of genome in the culture were calculated based on the copy number of 16S rRNA gene per genome, i.e., three in *C. aggregans* (NC_011831) and one in *C. thiodismutans* (Kojima et al., 2016).

### Sulfide and Sulfate Concentration Changes by *C. aggregans*-Dominated Microbial Mats

Microbial cell aggregates (microbial mats) that develop in hot spring water at 65°C at Nakabusa hot spring, Nagano, Japan were collected. The major member of these mats was *C. aggregans* as has been reported previously (Kubo et al., 2011; Everroad et al., 2012; Otaki et al., 2012). Approximately 1 g (wet weight) of the mats was placed into 70 ml glass vials containing 50 ml of a salt solution (1 mmol L^−1^ NaCl, 1 mmol L^−1^ Na_2_HPO_4_, pH 8.5) under N_2_ gas, and the vials were sealed with butyl rubber stoppers and aluminum seals. After pre-heating the vials at 65°C in the dark for 1 h, NaHCO_3_ and Na_2_S solutions were added to the vials (final concentration, 1 and 0.3 mmol L^−1^, respectively) and the vials were incubated at 65°C in the light (incandescent lamp; approximately 80 μmol⋅m^−2^⋅s^−1^) or dark. Sodium molybdenum oxide was added to the vial (final concentration, 20 mmol L^−1^) when indicated. Periodically 0.2 ml of the solution was collected from the vials for determining sulfide and sulfate concentrations in the cell-free supernatant, as described above.

## Results and Discussion

### Effects of Sulfide and Elemental Sulfur on the Growth of *C. aggregans* NA9-6

**FIGURE 1:**
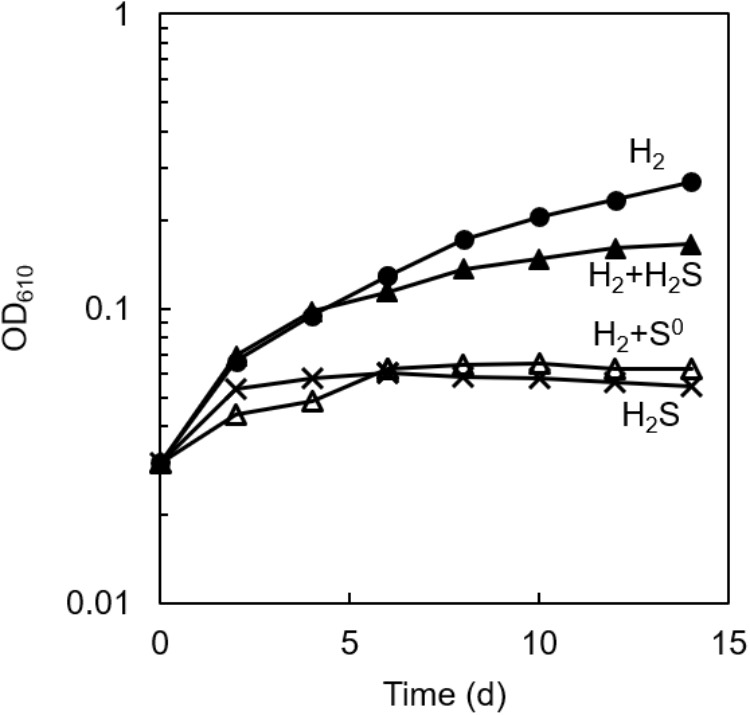
Effect of sulfur compounds on the autotrophic growth of axenic culture of *C. aggregans. C. aggregans* was cultivated in the autotrophic medium without sulfide and elemental sulfur (closed circle), 1 mmol L^−1^ of sulfide (closed triangles), 5 mg ml^−1^ of sublimed sulfur (open triangles) under H_2_:N_2_:CO_2_ (24: 56:20, v:v:v) atmosphere, and 1 mmol L^−1^ sulfide under N_2_:CO_2_ (80:20) atmosphere (crosses). Mean values of OD at 610 nm for three culture tubes are shown.

As shown in Figure 1, photoautotrophic growth was observed in the axenic culture of *C. aggregans* NA9-6 with H_2_ as the sole electron source (closed circles). However, no marked growth was observed with sulfide (crosses), and the addition of sulfide on the growth with H_2_ showed some inhibitory effect (closed triangles). No significant increase in OD was detected in the absence of sulfide and H_2_ (data not shown). From these observations, we supposed that the absence of the growth on sulfide as the sole electron source is possibly due to the early formation of elemental sulfur from the oxidation of sulfide by *C. aggregans*. This was partly supported by the observation that the H_2_-dependent growth was largely suppressed in the presence of sublimed sulfur (open triangles, Figure 1). Elemental sulfur has been known to inactivate sulfhydryl groups in enzymes (Libenson et al., 1953; Pestana and Sols, 1970), but its inhibitory mechanism for bacterial growth has not been elucidated yet. Abiotic production of polysulfide from elemental sulfur (Kamyshny, 2009) may also inhibit the sulfide-dependent growth by suppression of the enzyme reaction of sulfide:quinone oxidoreductase.

### Co-cultivation of *C. aggregans* and *C. thiodismutans* With Thiosulfate

**FIGURE 2:**
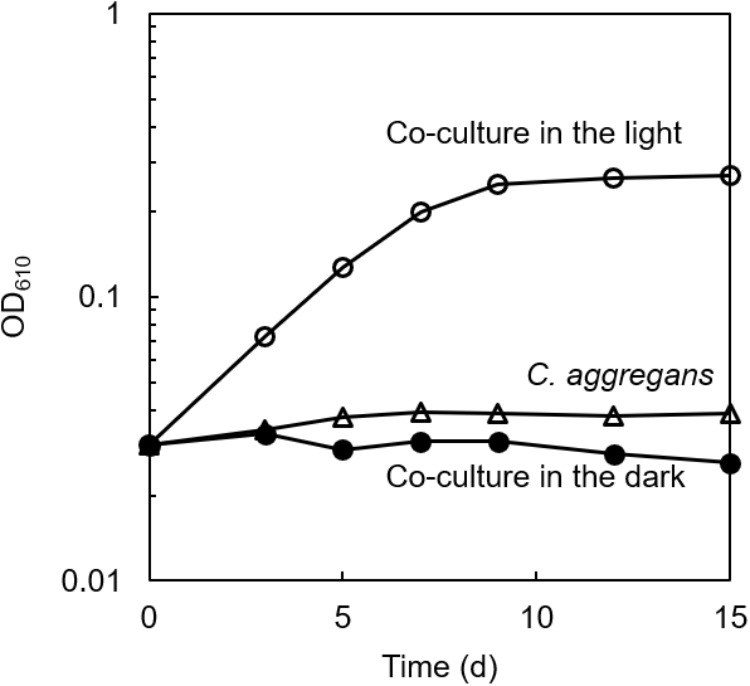
Symbiotic growth of *C. aggregans* and *C. thiodismutans* with thiosulfate in the co-culture. *C. aggregans* NA9-6 was cultivated in the autotrophic medium with thiosulfate under N_2_:CO_2_ (80:20) atmosphere with *C. thiodismutans* in the light (open circles) and in the dark (closed circles). Axenic culture of *C. aggregans* is also shown under the same conditions with open triangles. Mean values of OD at 610 nm for three culture tubes are shown.

The culture solution of *C. aggregans* NA9-6, cultivated under autotrophic conditions with H_2_ was mixed with the culture of *C. thiodismutans* TF1 that was cultivated in the presence of thiosulfate as the electron source and ferrihydrite as the sulfide scavenger. The mixture was cultivated in the autotrophic medium containing thiosulfate but without ferrihydrite under N_2_:CO_2_ (80:20) atmosphere. After more than 10 subcultures in medium without ferrihydrite, microscopic observation of the culture found no particles of ferrihydrite and iron sulfide. Only cells of both bacterial species, i.e., 200–300 μm length multicellular filamentous cells (*C. aggregans*) and 1.0–2.0 μm × 0.5–0.6 μm rods (*C. thiodismutans*) were observed (data not shown). Bacterial growth of the co-culture was evaluated by measurement of OD at 610 nm (Figure 2, open circles). OD increased almost exponentially during 8 days of cultivation and reached a maximum of 0.25 after that. The axenic culture of *C. aggregans* did not show such growth under the same conditions in the presence of thiosulfate (open triangles). No marked growth of the co-culture was observed in the dark (closed circles) and in the medium lacking thiosulfate or CO_2_ in the light (data not shown).

**FIGURE 3:**
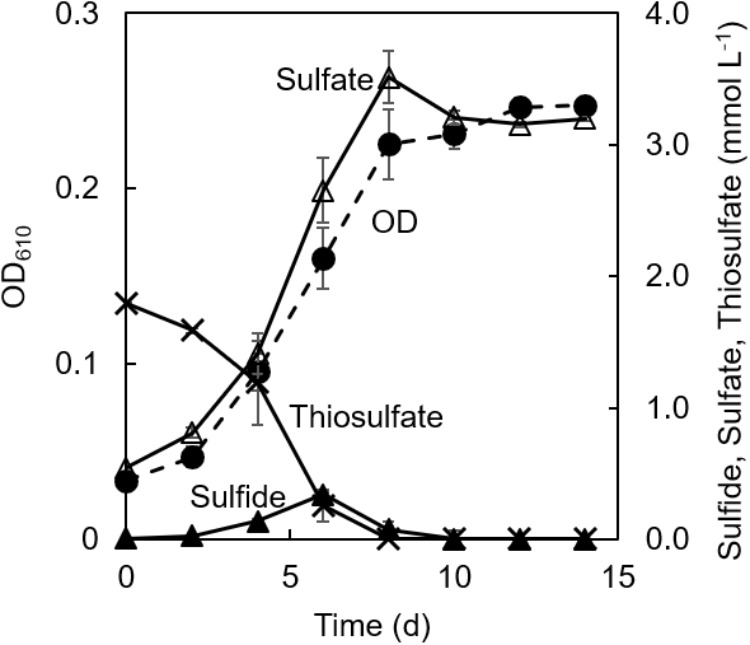
Changes of concentrations of sulfur compounds in co-cultivation of *C. aggregans* and *C. thiodismutans* with thiosulfate. Optical density at 610 nm of the co-culture (dashed line; closed circle), and concentrations of thiosulfate (solid line; crosses), sulfide (solid line; closed triangles), and sulfate (solid line; open triangles) in the culture supernatant are shown. The autotrophic medium contained thiosulfate and CO_2_. Mean values from three culture tubes are shown with standard deviations.

Figure 3 shows changes in concentrations of thiosulfate, sulfate, and sulfide in the co-culture along with the changes in the culture OD. Consumption of thiosulfate during growth was confirmed. Thiosulfate consumption was accompanied with the production of sulfate; approximately 1.8 mmol L^−1^ of thiosulfate was consumed and 3.1 mmol L^−1^ of sulfate was produced during 8 days of cultivation. 0.3 mmol L^−1^ of sulfide was detected at 6 days of cultivation, but the sulfide concentration was below 0.08 mmol L^−1^ after 8 days.

No accumulation of sulfide in the co-culture in the light suggested that *C. aggregans* grew using sulfide produced by *C. thiodismutans* as the electron source. *C. aggregans* worked as a biotic sulfide scavenger for *C. thiodismutans* through the photo-oxidation of sulfide, as previously described in co-culture of a sulfur-disproportionating bacterium in the genus *Desulfocapsa* with a purple sulfur bacterium in the genus *Lamprocystis* that oxidized sulfide to sulfate (Peduzzi et al., 2003). However, the role of the sulfur-disproportionating bacterium is different from the present study (described below).

### Co-cultivation of *C. aggregans* and *C. thiodismutans* With Sulfide

**FIGURE 4:**
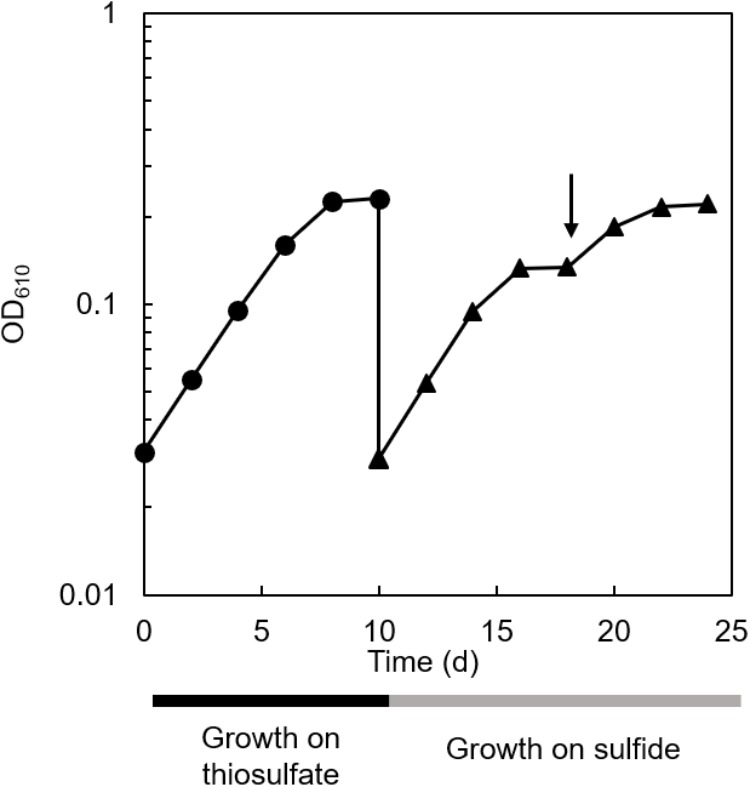
Sulfide-dependent growth of the co-culture after thiosulfate-dependent growth. Cultivation of the co-culture with thiosulfate (closed circles) and sulfide (closed triangles) as the sole electron source are shown. The arrow indicates the addition of 1 mmol L^−1^ sulfide.

The co-culture of *C. aggregans* NA9-6 and *C. thiodismutans* TF1 in medium containing thiosulfate (Figure 4, closed circles) was inoculated into medium containing 1 mmol L^−1^ sulfide but no thiosulfate and continuously co-cultivated (Figure 4, closed triangles). Residual thiosulfate was not expected at the time of inoculation as it was not detected at stationary phase as day 10 as shown in Figure 3. After inoculation with sulfide, immediate growth was observed suggesting sulfide metabolism had already proceeded in the culture with thiosulfate as indicated in Figure 3 and described above. After sulfide-dependent growth stopped 8 days after inoculation, sulfide was added again as indicated by the arrow in the figure, and additional growth was observed. Microscopic analysis indicated the growth of both bacterial strains (Supplementary Figure S1). No growth occurred in the absence of sulfide and thiosulfate (data not shown).

**FIGURE 5:**
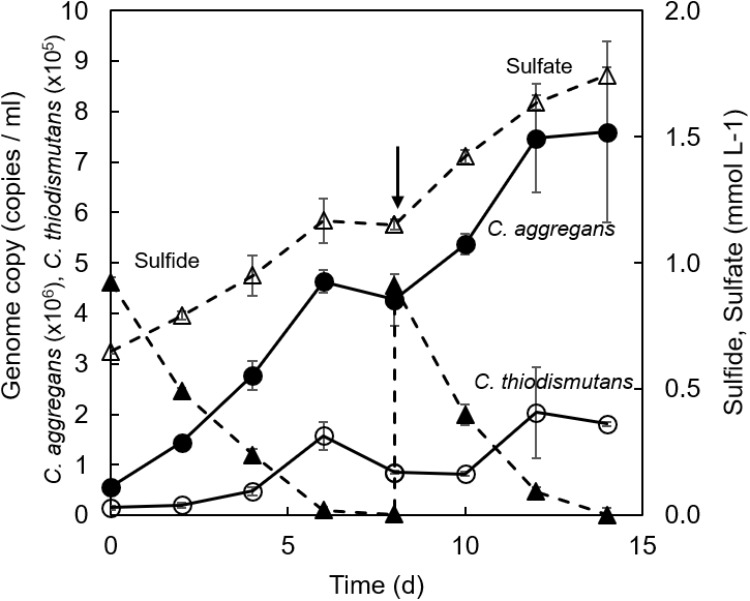
Changes of the number of genome copies and the concentrations of sulfur compounds in co-cultivation of *C. aggregans* and *C. thiodismutans* with sulfide. *C. aggregans* NA9-6 and *C. thiodismutans* TF1 were co-cultivated in medium containing sulfide. 16S rRNA gene copies for each species in the co-culture were determined by quantitative PCR to calculate the number of genome copies of *C. aggregans* (solid line; closed circles) and *C. thiodismutans* (solid line; open circles) in the culture solution. Sulfide (dashed line; closed triangles) and sulfate (dashed line; open triangles) in the culture supernatant were determined during the cultivation. The arrow indicates the point when 1 mmol L^−1^ sulfide was added into the culture. Mean values from three culture tubes are shown with standard deviations.

Growth of each bacterial strain in the co-culture was evaluated by quantitative PCR (qPCR) targeting the 16S rRNA gene of each strain after repetitive subcultures with sulfide. Figure 5 shows the qPCR results and the changes in concentrations of sulfide and sulfate in the culture. *C. aggregans* grew with the growth of *C. thiodismutans* during 6 days of cultivation. The growth of both strains was accompanied with the complete consumption of approximately 1 mmol L^−1^ sulfide and the accumulation of 0.5 mmol L^−1^ sulfate. No further growth was observed after 8 days, but the growth of both strains was observed again after the supplementation of 1 mmol L^−1^ sulfide into the culture. Ratio of (*C. aggregans* cells):(*C. thiodismutans* cells) was roughly calculated to be 30:1 ∼ 40:1 based on the qPCR results. This was consistent with microscopic observation (Supplementary Figure S1). These results indicate that *C. aggregans* fixed CO_2_ to grow in the co-culture.

The growth of *C. aggregans* under co-culture conditions indicated that *C. aggregans* grew autotrophically using sulfide as the electron source. The doubling time was 41.4 ± 0.9 h^−1^. This growth rate was approximately 2.5-fold higher than that of photoautotrophic growth using H_2_ in the axenic culture of *C. aggregans* (strain NA9-6) as shown in Figure 1. The growth of *C. thiodismutans* and accumulation of sulfate in the co-culture suggested that *C. thiodismutans* utilized elemental sulfur produced by *C. aggregans* as the electron source. *Chloroflexus aurantiacus* OK-70-fl which is phylogenetically close to *C. aggregans* grows photosynthetically with sulfide as the electron source, but lacks the ability to oxidize elemental sulfur (Klatt et al., 2007). Madigan and Brock (Madigan and Brock, 1975) reported in the culture of this bacterium that sulfur particles were observed around the cells with a microscope. In contrast, the deposited sulfur particles were not observed in the co-culture of *C. aggregans* with *C. thiodismutans* (Supplementary Figure S1).

*Chloroflexus* spp. are widely found in sulfidic hot springs and are known to possess genes for carbon fixation (Klatt et al., 2013; Weltzer and Miller, 2013). Recently, thermophilic sulfur-disproportionating bacteria were detected in various phylogenetic lineages from thermal environments including terrestrial hot springs (Mardanov et al., 2016; Thiel et al., 2017; Nishihara et al., 2018; Wilkins et al., 2019). However, their function *in situ* and interspecies interactions have not been examined yet. In the co-culture established in this study, *C. aggregans* likely produces elemental sulfur and the elemental sulfur seems to be simultaneously consumed by *C. thiodismutans*, whose population was 1/30 ∼ 1/40 of *C. aggregans*. Spatial proximity of these bacterial species may be related to their interactions through exchange of sulfur compounds in natural environments. An observation related to the spatial proximity was reported for purple sulfur bacteria that oxidized sulfide to sulfate and sulfur disproportionating bacteria at chemocline of a lake (Peduzzi et al., 2003; Tonolla et al., 2004, 2005). In natural environments, the both bacteria form cell aggregates together which are expected to increase the exchange efficiency of sulfur compounds and to overcome sulfide limitations. At Nakabusa hot springs, *C. aggregans* forms dense cell aggregates (microbial mats) with other bacteria which adhere to solid surface (Nakagawa and Fukui, 2002; Kubo et al., 2011; Everroad et al., 2012; Otaki et al., 2012) in sulfidic hot spring water.

We also examined the photo-oxidation of sulfide in *C. aggregans*-dominated microbial mats. *Chloroflexus*-dominated microbial mats were collected from under hot spring water at Nakabusa and a piece of the microbial mat was incubated anaerobically in artificial sulfidic hot spring water at 65°C. In the light, sulfide concentration gradually decreased with the increase in sulfate concentration within 12 h of incubation (Supplementary Figure S2a). The increase of sulfate was inhibited by the addition of molybdate (Supplementary Figure S2b) which is an inhibitor of dissimilatory sulfate reduction as well as sulfur-disproportionation (Taylor and Oremland, 1979; Finster et al., 1998). The addition of molybdate resulted in suppression of the sulfide consumption which was observed without molybdate in the light. These results suggest that elemental sulfur produced by photo-oxidation of sulfide was efficiently converted to sulfate and sulfide by elemental sulfur-disproportionation in the natural microbial mats.

**FIGURE 6:**
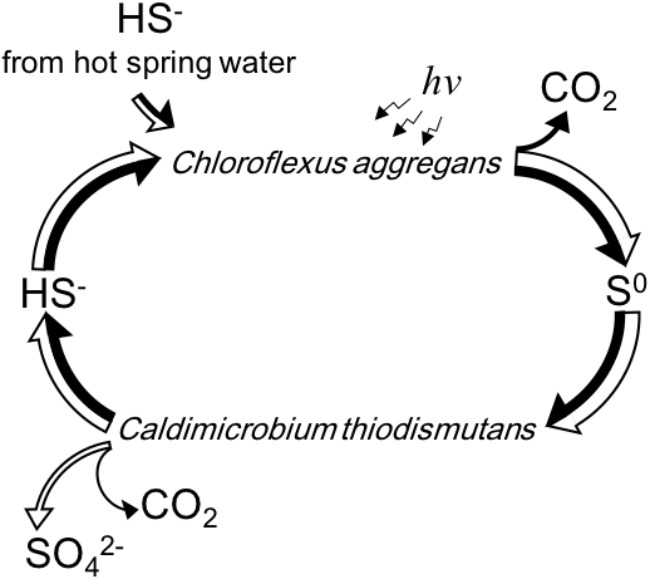
A schematic representation of the flow and cycling of sulfur (white arrows) and electrons (black arrows) mediated by *C. aggregans* and *C. thiodismutans in vitro* and *in situ* suggested in this study. The relative width of the axes of the arrows expresses the relative amount of the flow of sulfur or electrons approximately. Electrons are carried by the sulfur compounds except the flow to CO_2_ in the cells.

Figure 6 summarizes schematically the conversion of sulfur compounds and the associated electron flow in the novel symbiotic system with *C. aggregans* and *C. thiodismutans in vitro* with isolated strains as well as in microbial mats *in situ*. Sulfide is externally provided as the sole electron donor and *C. aggregans* converts it to elemental sulfur using light energy autotrophically. The elemental sulfur is used by *C. thiodismutans* with anaerobic chemolithoautotrophic growth producing sulfide and sulfate simultaneously through the process called disproportionation. The produced sulfide can then be used by *C. aggregans* again, and with all of these processes, externally provided sulfide is converted to sulfate by the symbiotic autotrophic system with two different autotrophic organisms performing photosynthesis and chemosynthesis. The electrons externally supplied with sulfide should mostly be used by *C. aggregans* to fix CO_2_ finally and only a few percent of electrons will be used by *C. thiodismutans* based on the observation that the amount of genome copies (Figure 5) and cells (Supplementary Figure S1) of *C. aggregans* was about 30 times larger than that of *C. thiodismutans* and the cell mass should be roughly parallel to the electron input to CO_2_. This explanation is consistent with the idea that most ATP is supplied by light-induced cyclic phosphorylation in *C. aggregans* and, on the other hand, ATP is produced by energetically unfavorable and electron-consuming process of the disproportionation of elemental sulfur to sulfide and sulfate in *C. thiodismutans*.

## Author Contributions

SK, NK, KM, and SH designed the study and analyzed and interpreted the data. SK performed the experiments in co-cultures. NK performed the experiments in natural microbial mats. SH supervised the experiments. SK, KM, and SH wrote and revised the manuscript. All the authors approved the final version.

## Conflict of Interest Statement

The authors declare that the research was conducted in the absence of any commercial or financial relationships that could be construed as a potential conflict of interest.

## References

[B1] Cline J. D. (1969). Spectrophotometric determination of hydrogen sulfide in natural waters. *Limnol. Oceanogr.* 14 454–458. 10.4319/lo.1969.14.3.0454

[B2] Everroad R. C. Otaki H. Matsuura K. Haruta S. (2012). Diversification of bacterial community composition along a temperature gradient at a thermal spring. *Microbes Environ.* 27 374–381. 10.1264/jsme2.ME11350 22673306 PMC4103544

[B3] Finster K. Liesack W. Thamdrup B. (1998). Elemental sulfur and thiosulfate disproportionation by *Desulfocapsa sulfoexigens* sp. nov., a new anaerobic bacterium isolated from marine surface sediment. *Appl. Environ. Microbiol.* 64 119–125. 9435068 10.1128/aem.64.1.119-125.1998 PMC124681

[B4] Giovannoni S. J. Revsbech N. P. Ward D. M. Castenholz R. W. (1987). Obligately phototrophic *Chloroflexus*: primary production in anaerobic hot spring microbial mats. *Arch. Microbiol.* 147 80–87. 10.1007/BF00492909

[B5] Hanada S. (2003). Filamentous anoxygenic phototrophs in hot springs. *Microbes Environ.* 18 51–61. 10.1264/jsme2.18.51

[B6] Hanada S. Hiraishi A. Shimada K. Matsuura K. (1995). *Chloroflexus aggregans* sp. nov., a filamentous phototrophic bacterium which forms dense cell aggregates by active gliding movement. *Int. J. Syst. Bacteriol.* 45 676–681. 10.1099/00207713-45-4-676 7547286

[B7] Holkenbrink C. Barbas S. O. Mellerup A. Otaki H. Frigaard N.-U. (2011). Sulfur globule oxidation in green sulfur bacteria is dependent on the dissimilatory sulfite reductase system. *Microbiology* 157 1229–1239. 10.1099/mic.0.044669-0 21233162

[B8] Holo H. Sirevag R. (1986). Autotrophic growth and CO_2_ fixation of *Chloroflexus aurantiacus*. *Arch. Microbiol.* 145 173–180. 10.1007/Bf00446776

[B9] Kamyshny A. (2009). Solubility of cyclooctasulfur in pure water and sea water at different temperatures. *Geochim. Cosmochim. Acta* 73 6022–6028. 10.1016/j.gca.2009.07.003

[B10] Klatt C. G. Bryant D. A. Ward D. M. (2007). Comparative genomics provides evidence for the 3-hydroxypropionate autotrophic pathway in filamentous anoxygenic phototrophic bacteria and in hot spring microbial mats. *Environ. Microbiol.* 9 2067–2078. 10.1111/j.1462-2920.2007.01323.x 17635550

[B11] Klatt C. G. Inskeep W. P. Herrgard M. J. Jay Z. J. Rusch D. B. Tringe S. G. (2013). Community structure and function of high-temperature chlorophototrophic microbial mats inhabiting diverse geothermal environments. *Front. Microbiol.* 4:106. 10.3389/fmicb.2013.00106 23761787 PMC3669762

[B12] Kojima H. Umezawa K. Fukui M. (2016). *Caldimicrobium thiodismutans* sp. nov., a sulfur-disproportionating bacterium isolated from a hot spring, and emended description of the genus *Caldimicrobium*. *Int. J. Syst. Evol. Microbiol.* 66 1828–1831. 10.1099/ijsem.0.000947 26842785

[B13] Kubo K. Knittel K. Amann R. Fukui M. Matsuura K. (2011). Sulfur-metabolizing bacterial populations in microbial mats of the Nakabusa hot spring, Japan. *Syst. Appl. Microbiol.* 34 293–302. 10.1016/j.syapm.2010.12.002 21353426

[B14] Libenson L. Hadley F. P. McIlroy A. P. Wetzel V. M. Mellon R. R. (1953). Antibacterial effect of elemental sulfur. *J. Infect. Dis.* 93 28–35. 10.1093/infdis/93.1.28 13069766

[B15] Madigan M. T. Brock T. D. (1975). Photosynthetic sulfide oxidation by *Chloroflexus aurantiacus*, a filamentous, photosynthetic gliding bacterium. *J. Bacteriol.* 122 782–784. 1092670 10.1128/jb.122.2.782-784.1975 PMC246117

[B16] Mardanov A. V. Beletsky A. V. Kadnikov V. V. Slobodkin A. I. Ravin N. V. (2016). Genome analysis of *Thermosulfurimonas dismutans*, the first thermophilic sulfur-disproportionating bacterium of the phylum *Thermodesulfobacteria*. *Front. Microbiol.* 7:950. 10.3389/fmicb.2016.00950 27379079 PMC4911364

[B17] Nakagawa T. Fukui M. (2002). Phylogenetic characterization of microbial mats and streamers from a Japanese alkaline hot spring with a thermal gradient. *J. Gen. Appl. Microbiol.* 48 211–222. 10.2323/jgam.48.211 12469320

[B18] Nishihara A. Thiel V. Matsuura K. McGlynn S. E. Haruta S. (2018). Phylogenetic diversity of nitrogenase reductase genes and possible nitrogen-fixing bacteria in thermophilic chemosynthetic microbial communities in nakabusa hot springs. *Microbes Environ.* 33 357–365. 10.1264/jsme2.me18030 30404970 PMC6307998

[B19] Otaki H. Everroad R. C. Matsuura K. Haruta S. (2012). Production and consumption of hydrogen in hot spring microbial mats dominated by a filamentous anoxygenic photosynthetic bacterium. *Microbes Environ.* 27 293–299. 10.1264/jsme2.ME11348 22446313 PMC4036054

[B20] Peduzzi S. Tonolla M. Hahn D. (2003). Isolation and characterization of aggregate-forming sulfate-reducing and purple sulfur bacteria from the chemocline of meromictic Lake Cadagno, Switzerland. *FEMS Microbiol. Ecol.* 45 29–37. 10.1016/S0168-6496(03)00107-7 19719604

[B21] Pestana A. Sols A. (1970). Reversible and mercurials and inactivation of rat by liver certain sulfhydryl elemental serine sulfur dehydratase enzymes. *Biochem. Biophys. Res. Commun.* 39 522–529. 10.1016/0006-291x(70)90609-1 5421952

[B22] Straub K. L. Kappler A. Schink B. (2005). Enrichment and isolation of ferric-iron- and humic-acid-reducing bacteria. *Methods Enzymol.* 397 58–77. 10.1016/S0076-6879(05)97004-3 16260285

[B23] Taylor B. F. Oremland R. S. (1979). Depletion of adenosine triphosphate in *Desulfovibrio* by oxyanions of group VI elements. *Curr. Microbiol.* 3 101–103. 10.1007/BF02602440

[B24] Thiel V. Hamilton T. L. Tomsho L. P. Burhans R. Gay S. E. Schuster S. C. (2014). Draft genome sequence of a sulfide-oxidizing, autotrophic filamentous anoxygenic phototrophic bacterium, *Chloroflexus* sp. strain MS-G (*Chloroflexi*). *Genome Announc.* 2:e00872-14. 10.1128/genomeA.00872-14 25189583 PMC4155588

[B25] Thiel V. Hügler M. Ward D. M. Bryant D. A. (2017). The dark side of the mushroom spring microbial mat: life in the shadow of chlorophototrophs. II. Metabolic functions of abundant community members predicted from metagenomic analyses. *Front. Microbiol.* 8:943. 10.3389/fmicb.2017.00943 28634470 PMC5459899

[B26] Tonolla M. Peduzzi R. Hahn D. (2005). Long-term population dynamics of phototropic sulfur bacteria in the chemocline of Lage Cadagno, Switzerland. *Appl. Environ. Microbiol.* 71 3544–3550. 10.1128/AEM.71.7.3544 16000760 PMC1169024

[B27] Tonolla M. Peduzzi S. Demarta A. Peduzzi R. Hahn D. (2004). Phototropic sulfur and sulfate-reducing bacteria in the chemocline of meromictic Lake Cadagno, Switzerland. *J. Limnol.* 63 161–170. 10.1016/S0168-6496(02)00354-9

[B28] van der Meer M. T. J. Schouten S. Bateson M. M. Nübel U. Wieland A. Kühl M. (2005). Diel variations in carbon metabolism by green nonsulfur-like bacteria in alkaline siliceous hot spring microbial mats from Yellowstone National Park. *Appl. Environ. Microbiol.* 71 3978–3986. 10.1128/AEM.71.7.3978-3986.2005 16000812 PMC1168979

[B29] Weltzer M. L. Miller S. R. (2013). Ecological divergence of a novel group of *Chloroflexus* strains along a geothermal gradient. *Appl. Environ. Microbiol.* 79 1353–1358. 10.1128/AEM.02753-12 23263946 PMC3568583

[B30] Wilkins L. G. E. Ettinger C. L. Jospin G. Eisen J. A. (2019). Metagenome-assembled genomes provide new insight into the microbial diversity of two thermal pools in Kamchatka, Russia. *Sci. Rep.* 9:3059. 10.1038/s41598-019-39576-6 30816235 PMC6395817

[B31] Zhu H. Qu F. Zhu L.-H. (1993). Isolation of genomic DNAs from plants,fungi and bacteria using benzy chloride. *Nucleic Acids Res.* 21 5279–5280. 10.1093/nar/21.22.5279 8255788 PMC310651

