# Supplementary figures and images for: Symbiotic Growth of a Thermophilic Sulfide-Oxidizing Photoautotroph and an Elemental Sulfur-Disproportionating Chemolithoautotroph and Cooperative Dissimilatory Oxidation of Sulfide to Sulfate
Source: Front Microbiol. 2019 May 24;10:1150. doi: 10.3389/fmicb.2019.01150 (PMC6543001; doi:10.3389/fmicb.2019.01150)

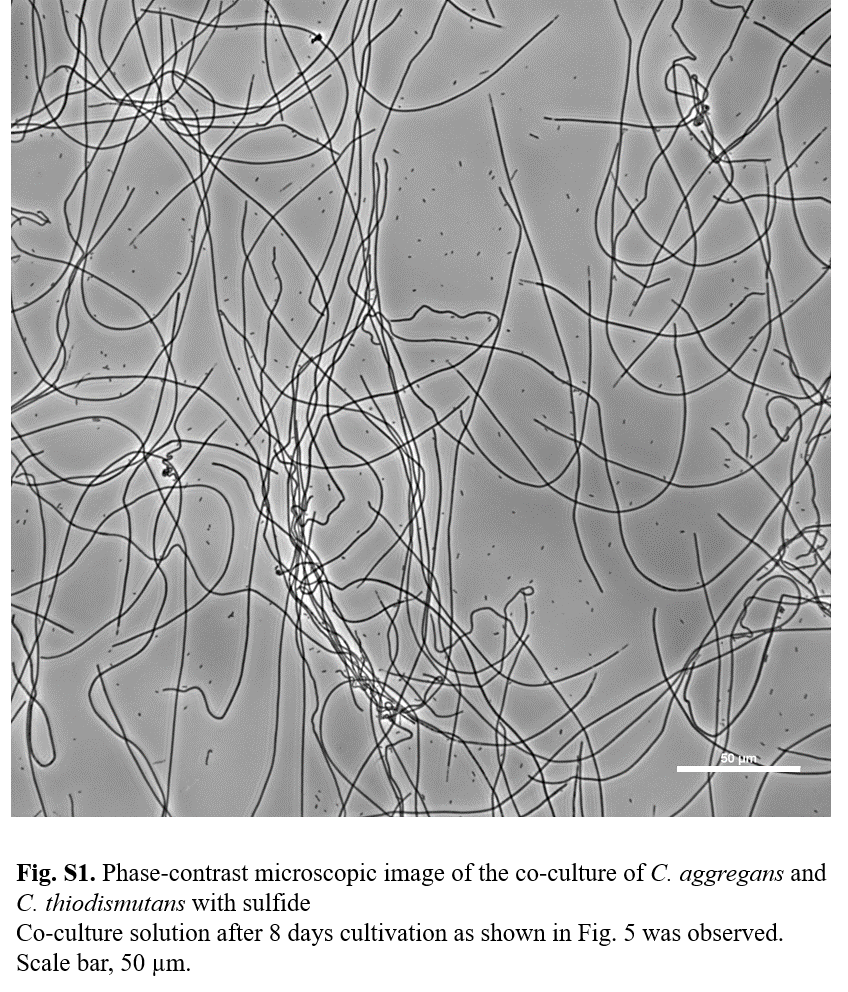

Supplement: Supplementary file 1 [file Image_1.TIF]

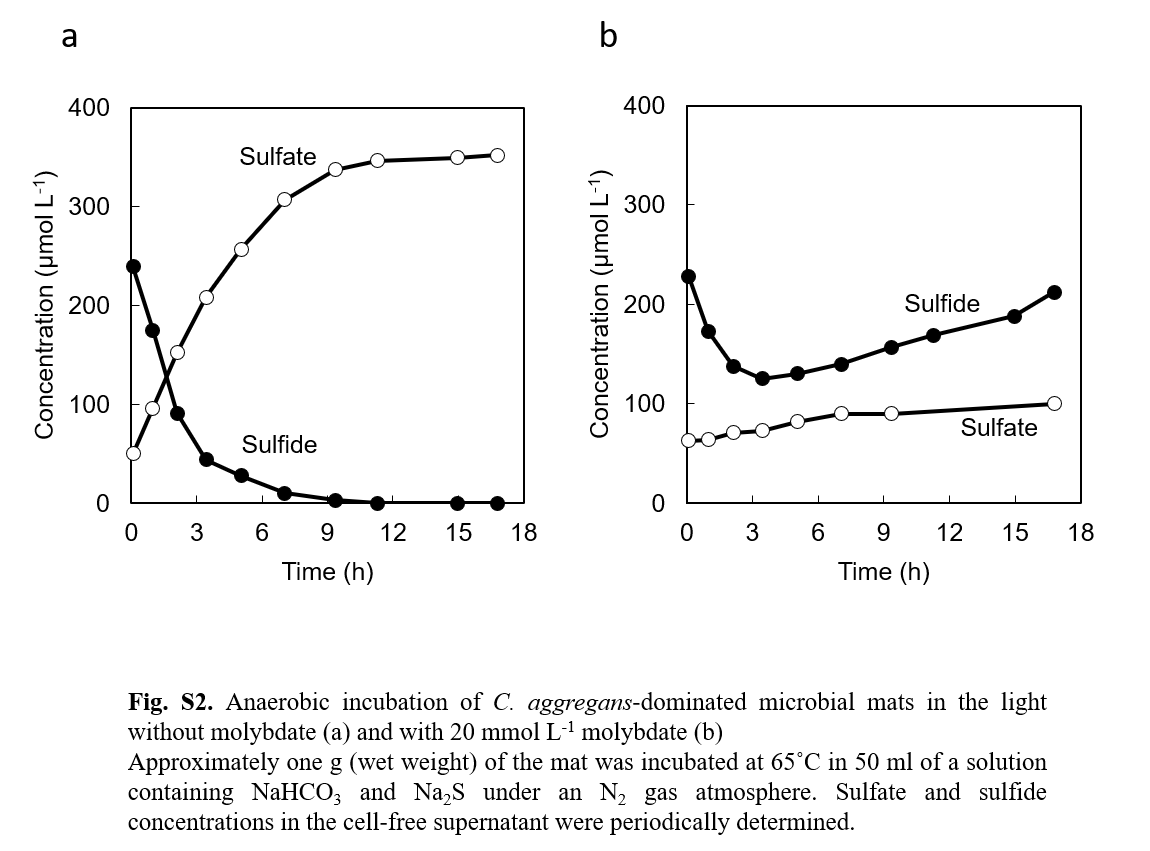

Supplement: Supplementary file 2 [file Image_2.TIF]
